# Ultrasonography- and Fluoroscopy-Guided Technique for Cooled Radiofrequency Ablation of the Genicular Nerves for Knee Joint Pain

**DOI:** 10.31486/toj.24.0038

**Published:** 2024

**Authors:** Swapnil Kumar Barasker, Anuj Jain, Sujeet Gautam, Dipti Saxena

**Affiliations:** ^1^ Department of Anesthesiology, Sri Aurobindo Medical College and Post Graduate Institute, Indore, India; ^2^ Department of Anesthesiology, All India Institute of Medical Sciences Bhopal, Bhopal, India; ^3^ Department of Anesthesiology, Sanjay Gandhi Postgraduate Institute of Medical Sciences, Lucknow, India; ^4^ Department of Anesthesiology, Super Specialty, MGM Medical College, Indore, India

**Keywords:** *Fluoroscopy*, *knee*, *osteoarthritis*, *pain*, *radiofrequency ablation*, *ultrasonography*

## Abstract

**Background:** Knee osteoarthritis is a chronic degenerative disease associated with pain and decreased mobility that affects advanced-age individuals, thus causing further debilitation. Radiofrequency ablation can benefit patients who are not ideal candidates for surgical intervention and for whom conservative management has been unsatisfactory. Currently, radiofrequency ablation is performed using either ultrasonography or fluoroscopy. In this technique review, we propose a method of performing cooled radiofrequency ablation of the genicular nerves that uses both ultrasonography and fluoroscopy and that could be helpful for novice pain practitioners.

**Case Series:** We report the experience of 2 patients with grade 4 osteoarthritis knee joints who underwent our cooled radiofrequency ablation technique. Each patient received a diagnostic block using ultrasonography, with ≥70% pain relief the prerequisite for performing cooled radiofrequency ablation. Our radiofrequency ablation technique involves using ultrasonography to identify and mark the superomedial, superolateral, and inferomedial genicular arteries. The marking done with ultrasonography is used during needle insertion with fluoroscopy guidance to reach the target points, and the final position of the needle is confirmed using sensory and motor stimulation before the cooled radiofrequency ablation procedure is performed. The cooled radiofrequency ablation resulted in pain reduction as measured on the visual analog scale and the Western Ontario and McMaster Universities Osteoarthritis Index scores at both patients’ 3- and 6-month follow-ups.

**Conclusion:** Using this technique for cooled radiofrequency ablation of the genicular nerves might help to reduce radiation exposure, specifically when the procedure is being performed by novice practitioners with limited experience.

## INTRODUCTION

Knee osteoarthritis is a leading cause of pain, disability, and morbidity in older individuals. In India alone, the prevalence of knee osteoarthritis is 28.7%; the numbers are higher in females and increase further with age progression.^1^ The definitive treatment for knee osteoarthritis is total knee replacement, but not all patients are willing to undergo the procedure or may not be ideal candidates because of comorbidities or advanced age. Among such patients, conservative management with nonsteroidal anti-inflammatory drugs, acetaminophen, and physiotherapy is the first line of therapy. Also recommended are intra-articular injections of steroids, hyaluronic acid, and platelet-rich plasma, but the efficacy of these therapies in patients with advanced knee osteoarthritis is questionable.^2^ Radiofrequency ablation (RFA) of the genicular nerves has emerged as a logical modality in patients with advanced osteoarthritis of the knee.^3,4^ Of the various ablation techniques, cooled RFA has shown the longest pain relief, from 12 months to 24 months.^5-7^

The major drawback with cooled RFA is the broader/wider cannula size (17 gauge) compared to the conventional RFA needle (20/22 gauge) that causes discomfort and pain in patients during cannula placement and at times requires conscious sedation and intravenous opioids.^8^ The common technique for needle placement is fluoroscopy, but the use of ultrasonography is increasing, as ultrasonography helps with identifying the genicular arteries and the genicular nerves. The genicular nerves are not directly visible with ultrasonography but are adjacent to the genicular arteries and thus form the needle target points.^9,10^ Ultrasonography-guided needle placement has been described in the literature,^10,11^ but the technique has drawbacks. During ultrasonography-guided procedures, novices prefer the in-plane needle technique because the out-of-plane technique requires expertise. But the in-plane technique is more painful because of the 17-gauge needle, and the needle requires a longer path to reach the target in comparison to the out-of-plane approach. Liberal use of local anesthetic is avoided during RFA because the anesthetic may alter the motor and sensory stimulation response required before RFA.

In this report, we suggest a needle placement technique that uses both ultrasonography and C-arm fluoroscopy that might improve the ease of performing the procedure and reduce radiation exposure, specifically when the procedure is being performed by novice practitioners.

## CASE SERIES

**Figure 1.**
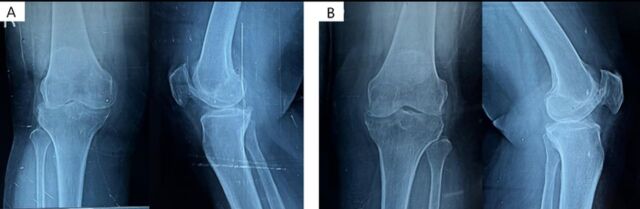
Anteroposterior and lateral views in the standing position for the (A) right knee joint of Case 1 and the (B) left knee joint of Case 2. Both x-rays show Kellgren and Lawrence grade 4 osteoarthritis, with severe joint space narrowing compromising the medial and lateral compartments, severe sclerosis, and multiple osteophytes.

The patients in this series had been managed conservatively with oral analgesics and physiotherapy, but both had poor pain relief and were planned for cooled RFA. We used the Kellgren and Lawrence scale to grade the patients’ knee osteoarthritis based on anteroposterior radiographs. The Kellgren and Lawrence grades range from 0 to 4, with grade 0 signifying no osteoarthritis and grade 4 signifying severe osteoarthritis.^12^ Both patients had grade 4 osteoarthritis. The patients’ knee x-rays are shown in Figure 1.

Baseline and postintervention pain severity were assessed with the visual analog scale (VAS) that ranges from 0 to 10, with 0 corresponding to no pain and 10 corresponding to worst pain.^13^ Baseline and postintervention pain, stiffness, and physical functions were assessed with the Western Ontario and McMaster Universities Osteoarthritis Index (WOMAC). WOMAC scores range from 0 to 96, with higher scores denoting higher levels of functional difficulty.^14^

The details of both cases are presented in the Table. The patients were admitted 1 hour before the procedure and were discharged on the same day 1 hour after the procedure. Consent for case publication was obtained from both patients.

**Table.** Patient Characteristics and Outcomes

			Pre-CRFA			Post-CRFA, 3 months		Post-CRFA, 6 months	
Case	Age, years/Sex	OA Grade^a^/Location	VAS	WOMAC	Diagnostic Block Relief^b^	VAS	WOMAC	VAS	WOMAC
1	78/M	4/Right knee	8/10	64/96	>70%	1/10	33/96	2/10	39/96
2	60/F	4/Left knee	7/10	62/96	>70%	2/10	32/96	3/10	39/96

^a^Assessed with the Kellgren and Lawrence scale, in which grade 0 signifies no osteoarthritis and grade 4 signifies severe osteoarthritis.

^b^≥2 hours.

Notes: The visual analog scale (VAS) ranges from 0 to 10, with 0 corresponding to no pain and 10 corresponding to worst pain. Scores for the Western Ontario and McMaster Universities Osteoarthritis Index (WOMAC) range from 0 to 96, with higher scores denoting higher levels of functional difficulty.

CRFA, cooled radiofrequency ablation; F, female; M, male; OA, osteoarthritis; VAS, visual analog scale; WOMAC, Western Ontario and McMaster Universities Osteoarthritis Index.

### Case 1

A 78-year-old male presented with complaints of pain in his bilateral knee joints for the past 15 years that had gradually increased in intensity. The right knee was more painful than the left knee, the medial side of the right knee joint was more painful than the lateral side, and at times the pain radiated to the patient's calf muscles. The patient was unwilling to undergo total knee replacement. He had a history of myocardial infarction 12 years prior and took aspirin 75 mg orally daily. His knee pain was managed conservatively with daily acetaminophen 500 mg every 12 hours orally, topical diclofenac-based gel, and occasional diclofenac 50 mg orally.

### Case 2

A 60-year-old female presented with pain in the left knee joint for the past 3 years. She reported that the pain increased on prolonged sitting and walking. The patient was managed conservatively with oral analgesics (acetaminophen 500 mg orally) and physiotherapy, but these therapies provided poor pain relief.

### Procedure Technique

#### Patient Position and Preparation.

Patients were placed in the supine position with the target knee slightly flexed 20° to 25° using a small bolster or rolled towel. A 22-gauge intravenous line was secured, and monitoring via electrocardiogram, pulse oximeter, and noninvasive blood pressure readings was performed during the procedure. Sterile painting and draping were done before performing the block.

#### Diagnostic Block Technique.

Each patient received an ultrasonography-guided diagnostic block on the target knee. The diagnostic block was given at 3 sites: 2 over the femur (superolateral and superomedial) and 1 over the tibia (inferomedial). Ultrasonography was performed using a high-frequency (6 MHz to 12 MHz) linear probe in the frontal plane, starting from the medial joint line for the superomedial and inferomedial genicular arteries. For the superomedial genicular artery, the probe was moved superiorly over the femur. For the inferomedial genicular artery, the probe was moved inferiorly over the tibia. Ultrasonography scanning was started from the lateral joint line for the superolateral genicular nerve and moved superiorly over the femur. The goal was to identify the genicular artery overlying the periosteum at the metaphyseal-diaphyseal junction of the femur and tibia. At the needle entry point, 1 mL l% lidocaine was injected in the subcutaneous plane. Using a 22-gauge, 10-cm Quincke spinal needle and the in-plane approach, 0.7 mL 2% lidocaine was deposited around the genicular arteries at all 3 target sites. Each patient was instructed to mobilize just after the block, and the block was considered successful if the patient's VAS decreased by ≥70% for at least 2 hours. Both patients had a successful block and were scheduled for cooled RFA.

#### Ablation Technique.

**Figure 2.**
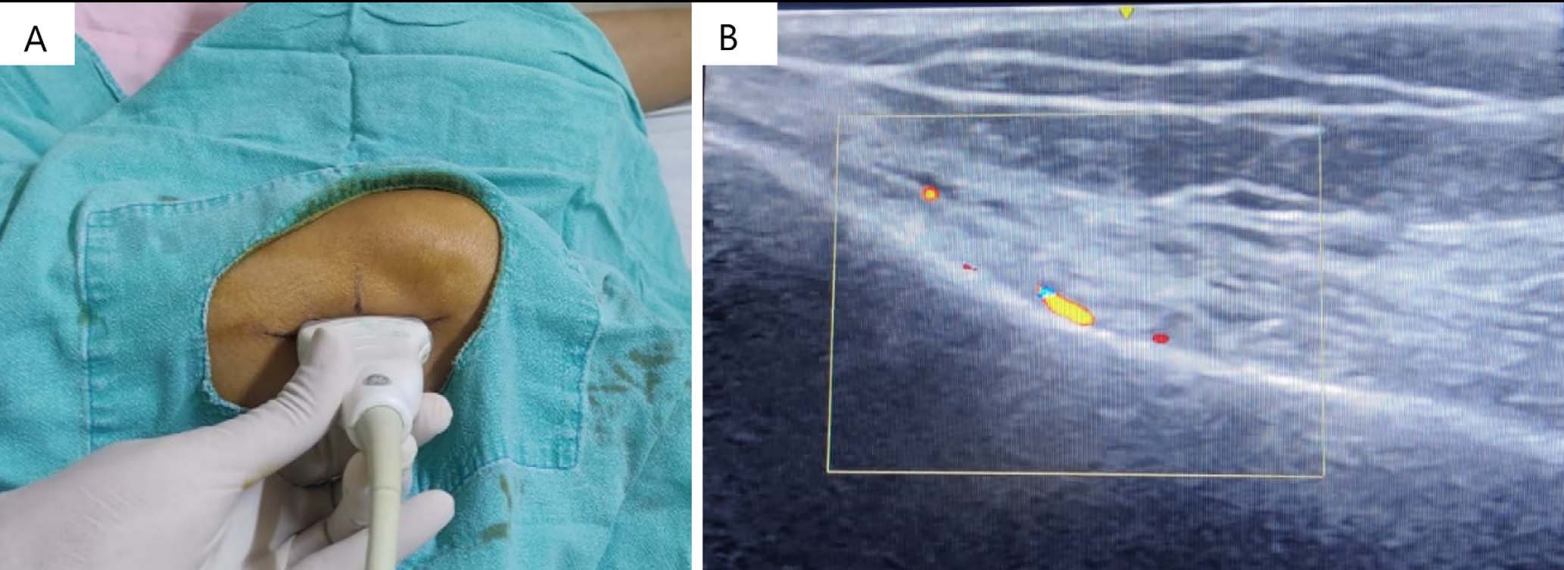
Ultrasonography scan for the right superolateral genicular artery. (A) The ultrasonography scanning is started from the lateral knee joint line, and the ultrasound probe is moved to the metaphyseal-diaphyseal junction of the femur superiorly, looking for the genicular artery overlying the periosteum. Once the genicular artery is visualized, the artery is centralized as per the center point mark on the ultrasonography machine screen. The ultrasound probe's central points are marked at 4 sites: 2 along the longitudinal axis and 2 along the transverse axis of the ultrasound probe. (B) Ultrasonography image with color scan of the superolateral metaphyseal-diaphyseal junction of the femur shows the centralized genicular artery with the center of the ultrasonography screen.

**Figure 3.**
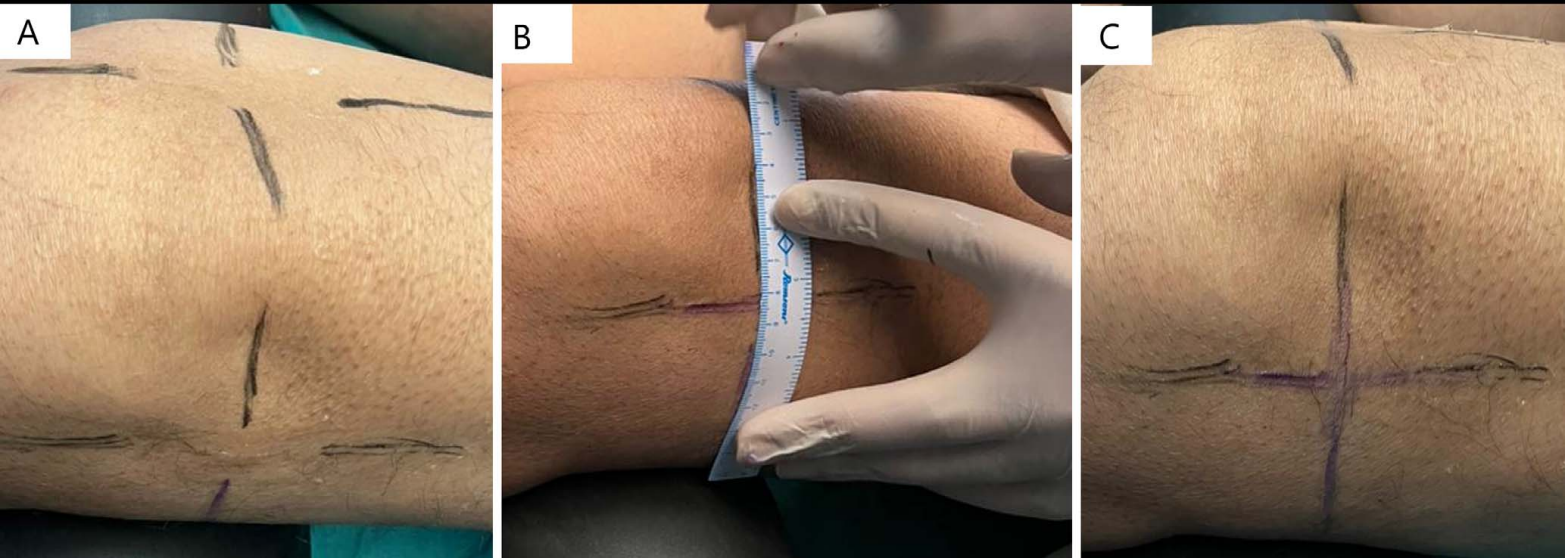
(A) The marked points after removal of the ultrasound probe. (B) The longitudinal and transverse marked points are joined using a ruler. (C) The joined lines have an intersection point.

The target genicular nerve sites were the superolateral, superomedial, and inferomedial for cooled RFA. The scanning technique was similar to that used for the diagnostic block. After localization of the genicular artery, the artery was centralized on screen with the corresponding central point mark on the ultrasound machine screen, and the ultrasound probe position points were marked on the skin as shown in Figure 2. After removing the probe, the lines were joined to form the intersection point of the transverse and longitudinal lines (Figure 3). Using color mode, the transverse line was scanned to identify any potential artery that the cannula could puncture during the procedure under fluoroscopy guidance. Sterile painting and draping were done after marking the probe points.

**Figure 4.**
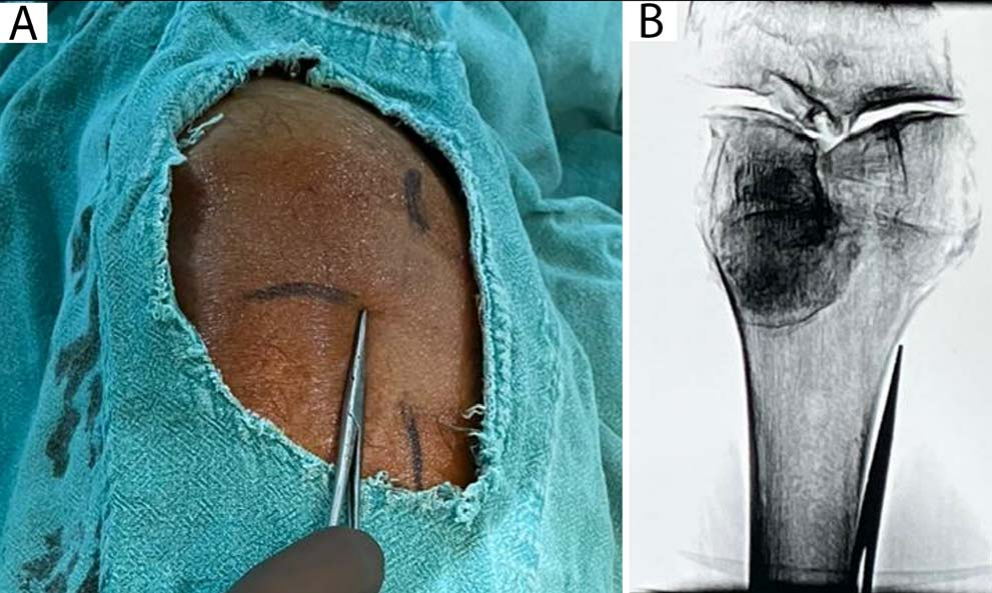
(A) A metal marker is moved over the transverse line to select the entry point. (B) The corresponding fluroscopy image shows the metal marker overlying the target area.

**Figure 5.**
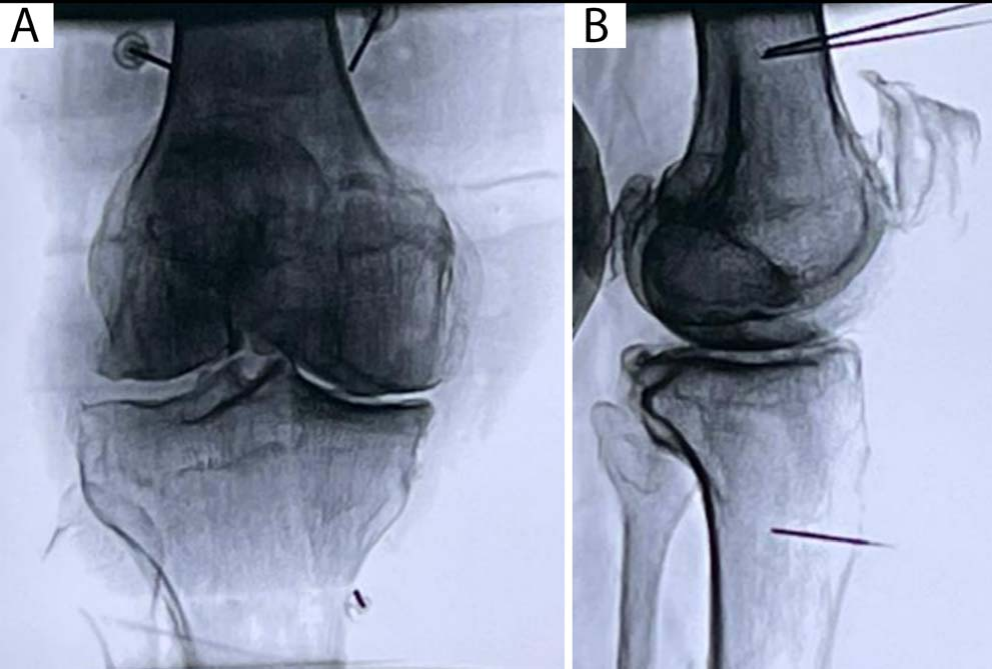
The final needle positions for the superolateral, superomedial, and inferomedial genicular nerves in (A) anteroposterior and (B) lateral views.

By moving a metal marker over the transverse line marked during ultrasonography, the confluent point of the metal marker and the bone was localized with fluoroscopy in the anteroposterior view. This point served as the entry point for the 17-gauge needle for cooled RFA (Figure 4). The distance between the entry point of the cooled RFA needle and the transverse and longitudinal line intersection point indicated the approximate depth of needle insertion required for ablation. After infiltration of local anesthetic (1% lidocaine) in the subcutaneous plane at the entry point, the needle was guided in line with fluoroscopy in anteroposterior view to contact the bone. The endpoint of cooled RFA needle insertion was determined in lateral fluoroscopy view, which was at approximately two-thirds to one-half the diameter of the lateral shaft of the femur or tibia (Figure 5).

The needle positions were considered successful if sensory stimulation was positive with concordant pain (same as the patient experiences) at 50 Hz and <0.5 mV and negative for any motor stimulations at 2 Hz and up to 2 mV. After successful stimulation, 2 mL 2% lidocaine was administered after negative aspiration through the cannula, 2 minutes prior to cooled RFA. Cooled RFA was done at 60 °C for 2 minutes 30 seconds each for the superolateral, superomedial, and inferomedial genicular nerves.

Using this technique, we did not have any challenges with successful stimulation. In both cases, we obtained positive sensory stimulation with concordant pain at 0.2 mV to 0.3 mV and 50 Hz at the target sites. We did not need to reposition the cannula for successful stimulation.

### Outcomes

Both patients tolerated the procedure without any discomfort and did not require any analgesics or sedation for the procedure. At consecutive follow-ups 3 months and 6 months after cooled RFA, the patients reported reduced consumption of analgesics (acetaminophen 500 mg) to a maximum of once to twice per week vs 2 to 3 times daily before the intervention. For both patients, scores on the VAS and WOMAC were considerably lower at both follow-up time points compared to the patients’ baseline scores.

## DISCUSSION

In the 2019 Guideline for the Management of Osteoarthritis of the Hand, Hip, and Knee, the American College of Rheumatology/Arthritis Foundation conditionally recommended RFA techniques for knee osteoarthritis.^2^ The recommendation was conditional principally because of the lack of a standard technique and the lack of long-term safety data. The development of a standard protocol or technique is difficult because of the variability in the anatomic landmarks for the genicular nerves.

RFA is generally performed under fluoroscopy guidance. Drawbacks to fluoroscopy are radiation exposure and the inability to visualize the genicular nerves/arteries, making the procedure dependent on operator skill. Ultrasonography can assist and at times replace fluoroscopy in the performance of regional procedures and blocks. Knee RFA using fluoroscopy or ultrasonography provides similar pain relief and functional improvement, but a benefit of ultrasonography is the reduced radiation exposure.^15,16^ The imaging modality choice also depends on the comfort level of the physician.^17^

**Figure 6.**
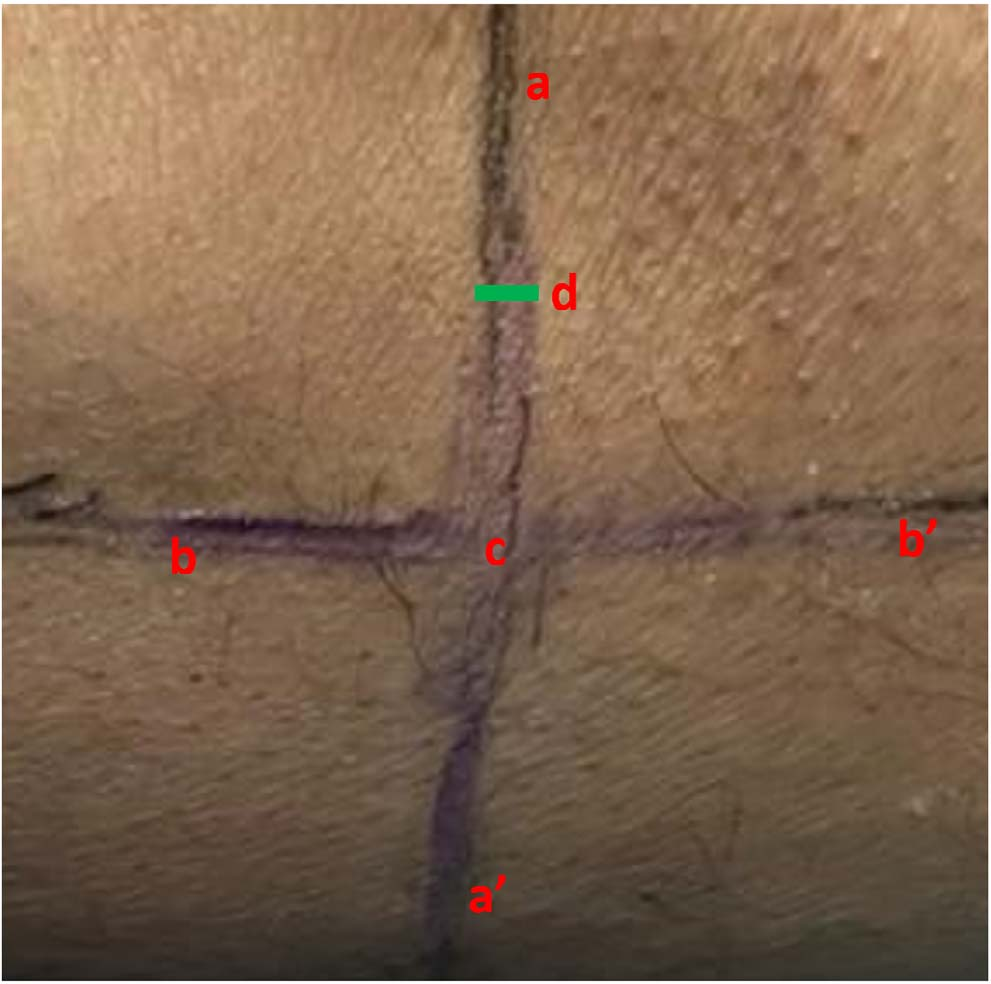
Determining the depth of needle insertion. The line from a to a′ is the transverse line. The line from b to b′ is the longitudinal line. The point of intersection of the 2 lines is denoted by c. The point of skin entry is denoted by d (green rectangle). This point is determined on anteroposterior fluroscopic view, at the junction of the metal marker over the bone as defined in Figure 4. The approximate required depth of needle insertion can be calculated by measuring the distance between d and c.

Advantages of our cooled RFA technique that uses both ultrasonography and fluoroscopy include the following:

1. The first step of successfully performing an ultrasonography-guided genicular nerve block is the visualization of the target point, the genicular artery. After visualizing the target, guiding the needle to the target requires good hand-eye coordination for stabilization of the probe and visualization of the full length of the needle in the in-plane approach.^17^ The challenges of hand-eye coordination can be overcome by using our technique as it does not require ultrasonography for needle positioning.
2. The entry point of the needle for performing the cooled RFA under fluoroscopy guidance is defined by the ultrasonography markings, so the need for fluoroscopy is reduced, leading to less radiation exposure than if the procedure were performed solely with fluoroscopy guidance.
3. The tentative path of the needle/cannula used for cooled RFA can be scanned using ultrasonography color mode which will lessen the risk of hematoma during performance of the procedure under fluoroscopy. Hematoma formation at the site of intervention is a rare complication of cooled RFA.^18^
4. Our technique provides a way to calculate the approximate depth required for the needle to reach the target genicular nerve (Figure 6). The depth can be confirmed by taking a lateral fluoroscopic view of the knee. The end point for the needle is at approximately two-thirds to one-half the diameter of the lateral shaft of the femur or tibia for the superomedial/superolateral and inferomedial genicular nerves, respectively. In our 2 patients, the depth of needle insertion corresponded with the measured length depicted in Figure 6.

In a 2023 cadaveric study, the genicular nerves were targeted using ultrasonography guidance to identify bony landmarks. The authors of the study found that even in the absence of arterial pulsations, genicular nerve block could be performed with 100% accuracy.^11^ Targeting genicular nerves in the absence of genicular artery visualization with ultrasonography and using bony landmarks are topics of future research that have the potential to help patients in whom identifying the arterial pulsations of genicular arteries is difficult.

Limitations of this case series are the limited number of patients and the short-term 3- and 6-month follow-up. We do not have data on the duration of pain relief.

## CONCLUSION

The cooled RFA procedure using both ultrasonography and fluoroscopy for osteoarthritis of the knee has the potential benefit of reduced radiation exposure. Studies with a prospective design and long-term follow-up will help elucidate the potential of this technique, as well as any drawbacks.

## Notes

*This article meets the Accreditation Council for Graduate Medical Education and the American Board of Medical Specialties Maintenance of Certification competencies for Patient Care and Medical Knowledge.*
